# Quantifying the realistic reduction potential of food waste in Swedish households

**DOI:** 10.1038/s41598-026-37302-7

**Published:** 2026-02-02

**Authors:** Amanda Sjölund, Niina Sundin, Erik Svensson, Yvette Paula Aroko, Mattias Eriksson, Christopher Malefors

**Affiliations:** Department of Energy and Technology, Swedish University of Agricultural Sciences, Box 7032, SE-75007 Uppsala, Sweden

**Keywords:** Food systems, Impact assessment, Sustainable development goals, Food waste prevention, Climate sciences, Ecology, Ecology, Environmental sciences, Environmental social sciences

## Abstract

Household food waste is often framed as a critical challenge for food systems, yet the real potential and impact of waste reduction remain uncertain. This study assessed the prevention potential and the carbon, economic and nutrition footprints of food waste in 41 Swedish households, using data from a digital quantification system over a total of 9843 days. Results showed that 24.4% of food waste was avoidable and 7.3% was possibly avoidable, indicating a prevention potential of 31.7% in total food waste. These fractions had a joint carbon footprint of 19 kg CO_2_e and an economic cost of €66 per person per year. Despite its smaller volume, the possibly avoidable fraction entailed notable nutrient losses, indicating a missed opportunity for improved nutrition. Additionally, two scenarios modeling a 50% reduction in food waste indicated limited climate, economic, and nutritional benefits from halving food waste. These findings suggest that the impact from reducing food waste among Swedish households may be more limited than typically portrayed. Efforts and resources directed toward reducing food waste should therefore be weighed against, or combined with, other interventions, such as promoting dietary shifts, that may offer greater benefits for food system transition.

**Supplementary Information:**

The online version contains supplementary material available at 10.1038/s41598-026-37302-7.

## Introduction

Global food systems face significant challenges that are impeding the ambitions of sustainable development. Along with current dietary patterns and agricultural practices, food waste has been identified as a critical challenge requiring action to reverse the unsustainable trend within food systems^1–3^. Reducing food waste has therefore become increasingly recognized by researchers, governments, and organizations as one of the key stepping stones to reach a sustainable food system as this would mitigate environmental impacts, reduce financial losses, and save nutrients^4–6^.

Recognizing food waste as a major global issue, various institutions have set targets to address this, with the most widely acknowledged being Target 12.3 of the United Nations’ Sustainable Development Goals (SDGs). This target calls for a 50% reduction in food waste at the consumer level by 2030^7^, including households which is estimated to be a leading source of food waste along the supply chain^8^. In response, governments and organizations worldwide have established goals to align with the SDG commitment. Within the European Union (EU), a general directive has been in place since 2018, urging member states to implement measures that promote food waste reduction in accordance with SDG 12.3^9^. However, a specific reduction target for member states to aim towards has been lacking. Therefore, in 2023, the European Commission proposed a legally binding target requiring member states to reduce food waste by 30% across retail, restaurants, food services, and households^10^. This proposal indicates a growing commitment to address food waste, and a call for resources to be directed towards research, policies, and initiatives aimed at meeting these ambitious objectives.

However, there is a lack of guidelines about how such targets can be achieved and how to combine policy measures for food waste reduction with other policy objectives^11^. Moreover, the benefits that can be achieved through food waste reduction are not simply determined by the quantity of waste reduced but also by the type of food wasted^12,13^. In this regard, targets also remain unclear about the definition of “food waste” and what type of food waste should be included, if it is only the avoidable fraction or all food waste^14^. At present, there is no defined distinction between avoidable and unavoidable food waste which has caused uncertainties around how food waste is quantified and categorized^15^. This resultingly hampers comparability across studies and complicates the assessment of the prevention potential which is necessary to guide interventions^16^.

Aside from uncertainties in definitions, food waste estimates are further affected by the lack of primary data and standardized quantification methods^17,18^. Even when primary data is available, the quantification methods used to track food waste levels in households have been repeatedly questioned regarding their ability to provide reliable assessments^19,20^. Commonly used methods include questionnaires and food waste diaries where households self-report on their food waste either by estimation or by tracking their waste using scales. However, because these methods are influenced by subjectivity and depend on the households’ commitment to engage in the task, reported food waste amounts are often underestimated^21^. In contrast, approaches such as waste composition analyses and waste audits are performed by third parties that collect the food waste which provides more objective estimates. However, these are resource-intensive and costly methods which ultimately limit their ability to be applied across larger samples and over longer periods^22^. The difficulty in monitoring food waste in households over longer time periods, i.e., longer than a few weeks, is a pronounced limitation of most quantification methods since they require manual input. In an attempt to address the lack of long-term monitoring abilities, there have been initiatives to apply more technological solutions that can track food waste over time without relying on manual labor^23^. However, such technologies are not yet deployed at any larger scales at the household level.

The uncertainties in food waste data are arguably reflected in assessments of household food waste where, only in Europe, the discrepancy between countries’ statistics on how much of total food waste could be avoided is substantial. For example, in Sweden, official food waste statistics suggest that 27% of food waste in households is avoidable^24^, whereas in the United Kingdom, the corresponding figure has been reported to be approximately 70%^25^. Although food waste quantities and patterns can vary between households, cultures, and countries, these factors alone seem unlikely to account for the considerable difference between the two countries. Instead, the difference is more plausibly attributed to methodological factors, such as the uncertainties in applied quantification methods. The lack of reliable quantification methods, and consequently, reliable estimates, raises fundamental questions about the true prevention potential of food waste in households. Without information on how much food is wasted and how much of it could realistically be avoided, it is difficult to determine the scale of the issue and how to prioritize actions. This also presents further considerations, such as the benefits that can be expected if reduction targets are achieved. It also becomes difficult to assess potential trade-offs that might require consideration in regard to interlinkages between e.g. economic and environmental impacts^26^.

Previous studies that have assessed the consequences of household food waste have also shown that different metrics yield varying outcomes, highlighting the complexity in the problem. Using the impact of different food categories as an example, it has been found that by weight, the greatest reduction potential lies in fruits and vegetables, although these have less potential for economic savings and climate impact mitigation compared to animal-based food waste^12,13^. On the other hand, the greater amount of fruit and vegetable waste infers a loss of nutrients that are frequently under-consumed. Therefore, reducing waste of this fraction may be more beneficial from a nutritional point of view^27–29^. These contradictory implications indicate that the benefits of reducing food waste might depend on what type of food waste is prevented as well as the perspective taken. Nevertheless, although the results of previous studies largely agree on the benefits that can be achieved by preventing food waste, questions persist about how significant the potential for prevention is, what data or measurements are supporting these claims, and what impact a reduction in food waste levels could generate.

Considering these questions, the aim of this study is to analyze long-term food waste data from Swedish households, focusing on its quantity and composition, and to assess its prevention potential. Furthermore, the study evaluates the climate, economic, and nutritional impacts of food waste, and explores the potential benefits across these dimensions of reducing food waste by half.

## Materials and methods

To meet the study objectives, the work was divided into three steps: (i) determining the quantity and composition of the food waste; (ii) assessing the prevention potential of the wasted food; and (iii) assessing the impact of preventable food waste and the potential benefits of reducing it by half. All work was performed in accordance with relevant guidelines and regulations; ethical approval was not required under the Swedish Ethical Review Act (2003:460)^30^, and informed consent was given by all participants.

### Food waste quantification

Food waste data was collected from 41 Swedish households (93 individuals) that had monitored their food waste for a total of 9843 days during different periods between April 2023 and January 2025, using a digital quantification system. Information about the households and the data collected is available in the Supplementary material (Table S1). The quantification system, described in more detail in Sjölund et al.^31,32^, consists of a scale that automatically records the weight of food waste items as they are disposed of and a camera positioned above the scale which captures an image of the wasted items. Weight and image data for each waste event are recorded by a single-board computer that uploads all data to a server for central storage and backup.

**Fig. 1:**
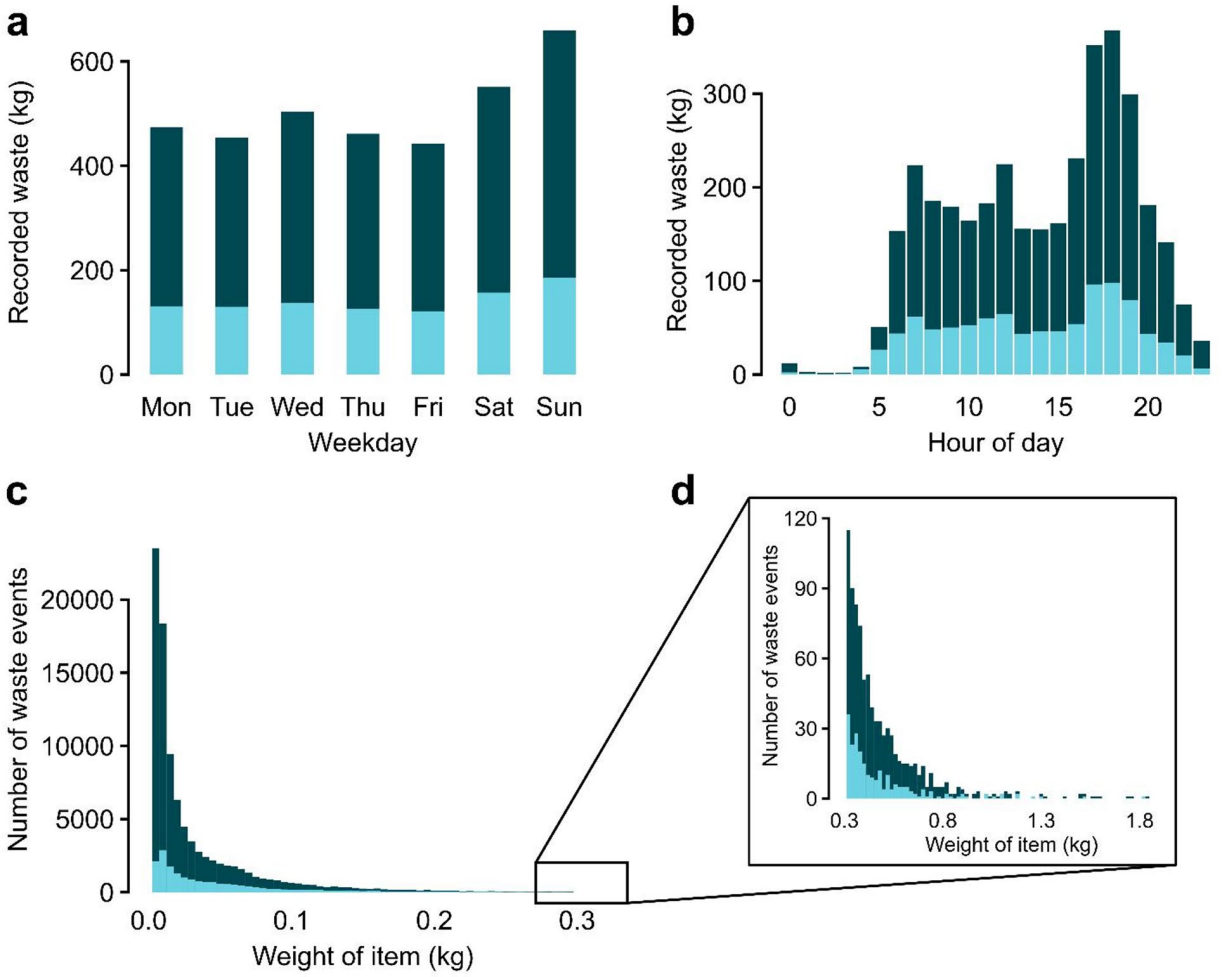
Distribution of recorded food waste, divided into uncategorized (), and categorized () events. (**a**) displays the distribution of total recorded weight between days, and (**b**) between hour of the day. (**c**) displays the distribution of waste items with weights up to 0.3 kg, and (**d**) displays items between 0.3 and 2 kg.

Throughout the monitoring period, a total of 94,248 waste events were recorded, totaling 3546 kg of food waste. Of these, 17,358 events with viable data (919 kg) were categorized, excluding images that were too dark, events where the content of the food waste bin was not visible, and instances of measurement or image errors. To confirm that the categorized events were representative of the uncategorized events, the two groups were compared based on the time of recording and weight distribution. As shown in Fig. 1, both groups followed a similar pattern, indicating that the categorized data provided a fair reflection of the overall recorded food waste.

### Categorization

**Fig. 2:**
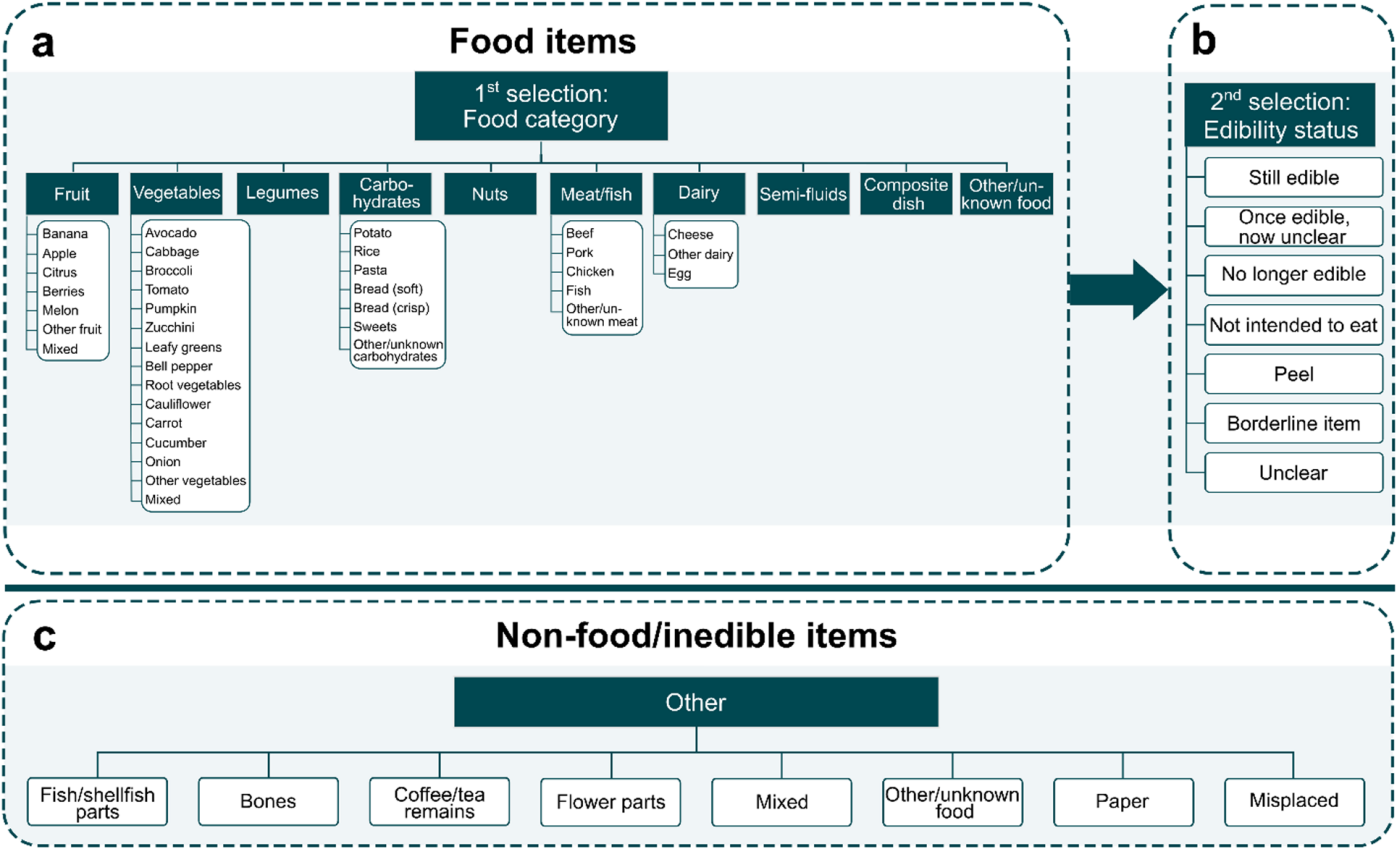
Categorization framework used to determine the composition of food waste. (**a**) shows the included food categories which were then assigned an edibility status as listed in (**b**). If an item was not applicable to categorize according to items in (**a**), it was assigned the appropriate category listed in (**c**).

The categorization of food waste was performed manually by two of the authors using a custom-built online interface designed for this study. The selected food waste categories were inspired by previous studies (e.g., WRAP 2023; Sigala et al. 2024), but adapted to a Swedish context to include foods commonly consumed in Sweden. In the categorization process, before-and-after images of waste events were randomly selected from the database and displayed on the interface. The authors then assigned each event to the appropriate food category displayed in Fig. 2.

The categorization framework was structured to first identify the food item visible in the afterimage, which displayed the discarded waste of one recorded event. If the item matched a category listed in Fig. 2b, it was selected accordingly. When applicable, a subcategory (white boxes in Fig. 2a) was also specified, for example, within the vegetables and fruit categories. Next, the edibility status of the selected item was determined (Fig. 2b) as either avoidable or not intended for consumption (i.e., unavoidable). The unavoidable category included items such as peels and stones/cores of fruit and vegetables, eggshells, fish and meat bones/skins, nut shells, and the top/bottom parts of vegetables. However, peels that could have been consumed, such as those of potatoes, carrots and apples, were not considered unavoidable and were instead categorized separately under “Peel”. Similarly, given that perceptions of what is avoidable vary across individuals, studies, and cultures, a separate “Borderline item” category was included. This category included items such as broccoli stalks and cauliflower leaves which were considered possible to eat but often discarded. The categories including peels and borderline items were later grouped under the joint edibility status “possibly avoidable” food waste.

Moreover, because a single waste event could include multiple food items, more than one food item listed in Fig. 2a could be selected for each event. However, only one edibility status (Fig. 2b) could be assigned per event to ensure consistency. In cases where multiple items with differing edibility status were present, the edibility status was determined based on the item contributing the most weight. If this was unclear, the “Mixed” option in Fig. 2c was selected.

#### Treatment of data

The categorized food waste was initially processed in Microsoft Excel to ensure that each wasted item was assigned a separate weight. When multiple food items had been categorized as “mixed”, images were re-examined to manually assign them categories according to the framework. This procedure redistributed mass across categories but did not affect the total amount of recorded food waste. Additionally, when multiple items had been discarded in the same waste event, resulting in multiple food items having been selected at the categorization stage, their total weight was evenly distributed among the items.

When all recorded food waste had been divided into single items, these were then separated into three fractions: avoidable, possibly avoidable, and unavoidable. The avoidable fraction included all items categorized as “still edible”, “once edible, now unclear”, and “no longer edible”. The possibly avoidable fraction was comprised of food items sorted under the edibility statuses “peel” and “borderline item” (Fig. 2b). The unavoidable fraction included items categorized as “not intended to eat” as well as items sorted into the “other” category listed in Fig. 2c, except for the “mixed” category which was included in the avoidable fraction. To improve clarity and coherence, the avoidable food categories were consolidated into eight main categories. Additionally, to assess the prevention potential, both avoidable and possibly avoidable fractions were considered and were therefore grouped into the joint fraction preventable food waste.

Following the initial processing, the dataset was imported to R to generate descriptive statistics. Since not all generated food waste had been categorized, the study assessed the prevention potential using the collective average across the entire dataset (which also included non-categorized food waste) rather than evaluating each household individually. Further, to contextualize the results, a point of reference was set to *kg food waste per person per day*, based on average data. Since the digital quantification system only captured days when food waste was recorded, while in reality food waste is not generated every day, the daily average was adjusted to account for this by assuming that in 14% of days, no food waste occurs, as suggested by Sjölund et al.^32^. This allowed for a more accurate daily estimate, which could then be scaled to an annual estimate by multiplying by 365, without the risk of overestimating the aggregated estimate.

### Impact assessment

To assess the impact of food waste, assessments in terms of climate, economic, and nutritional impacts were conducted. The impact assessments focused exclusively on the preventable food waste fraction as it represents the portion that could realistically be prevented through changes in behavior and more efficient food use. Including the unavoidable fraction was considered to overstate the potential for reduction and ultimately misrepresent the benefits that could be achieved.

#### Climate impact assessment

The climate impact was assessed by calculating the carbon footprint in carbon dioxide equivalents (CO_2_e) using the Sustainability Assessment of Foods and Diets (SAFAD) tool^34^ (described in more detail in^35^, which provided carbon footprint values for each food waste item. Each subcategory within the categorization framework was assigned a carbon footprint corresponding to that category. For categories containing different possible food items (e.g., cheese), an average carbon footprint was calculated based on reasonable options within that category.

For composite dishes, images from the categorized events were re-examined to assign dish-specific carbon footprints from the SAFAD tool. The total carbon footprint of composite dishes was then divided by the category’s total weight to assign an overall category-specific carbon footprint. The same approach was used for the semi-fluids category. Additionally, for sub-categories labelled as “other” under the fruit, vegetable, carbohydrate, meat/fish, and dairy categories, an average carbon footprint of other commonly consumed foods in Sweden was assumed for each category. Similarly, items under the sub-category “mixed” were assigned the average of the corresponding category. Events that were only categorized as “mixed” without a specified food category (Fig. 2c) were assigned the overall average carbon footprint across all categories. Finally, since the SAFAD tool did not have carbon footprints for specific parts of food items (e.g. potato peels), the carbon footprints of the whole item (e.g. potatoes) were used. A detailed breakdown of all categorized items and their assigned carbon footprints is available in Table S2 in the Supplementary material. The total carbon footprint for each fraction, avoidable and possibly avoidable, respectively, was then divided by the total weight of the food waste to obtain a carbon footprint per kg of wasted food.

In the SAFAD tool, the carbon footprint of food items includes all stages of the supply chain until consumption, meaning that the carbon footprint of the waste management is not included. To account for the carbon footprint of managing the wasted food, anaerobic digestion was considered as the waste treatment option. This aligned with both the local waste treatment practices in the studied households and Swedish targets to increase source separation of food waste and enhance material recovery through anaerobic digestion and composting^36^. This is also how the quantified food waste gets treated since the methodology only captures the waste that is sorted into the organic waste bin. To derive an average value of the climate benefits from the waste management process (anaerobic digestion), values from Feiz et al.^37^ were used, suggesting a climate impact of -0.10 kg CO_2_e per kg food waste.

#### Economic impact assessment

The economic impact assessment was conducted from a household perspective, focusing on the direct costs paid by the households. Costs related to the digital quantification system were excluded as the households did not bear these expenses, apart from electricity usage, which was considered negligible. To estimate the monetary losses associated with the wasted food, the cost of food items from the online shop of the largest retailer on the Swedish market was used^38^. Average prices of common brands and products available in February 2025 were used to approximate the cost of each food category.

For pasta and rice, which were usually cooked prior to being wasted, prices were converted into cooked weight by assuming that half of the cooked weight of pasta and two-thirds of rice was water. For composite dishes, pricing was based on ingredient lists from online recipes and restaurant menus. If a meal appeared to be homemade (e.g., stews and pancakes), the ingredient cost of recipes for reoccurring dishes from the images were used to obtain an average price for a composite meal. To reach cost per kg, an average portion size of 400 g was assumed. For composite dishes that appeared to originate from restaurants (such as pizza or hamburgers) or were purchased as ready-made meals from the supermarket, corresponding restaurant and supermarket prices were assumed. The cost of preparing food, including electricity costs, were excluded from the assessment. The total cost was then divided by the total weight of the wasted food to acquire an average cost per kg of food waste. All prices and costs originally recorded in Swedish Krona (SEK) were converted to Euro (€) using an exchange rate of SEK 11 per €.

In Sweden, food waste is collected as a separate waste fraction with households paying a fee for the municipality to collect their food waste. Allocated to purely the collection of food waste, households in the municipality where most study participants live pay an annual fee of approximately €70 to the municipal waste management services^39^. However, because the fee for waste management collection is a fixed cost and independent of the amount of generated food waste, the cost for waste management was excluded from all economic assessments.

#### Nutritional impact assessment

The nutrient losses associated with avoidable and possibly avoidable food waste were calculated using Nutrition Data^40^ software and food composition data from the Swedish Food Agency^41^ along with food composition data from the United States Department of Agriculture^42^. The total energy, macronutrient, micronutrient, and dietary fiber contents of the waste were determined for the entire data collection period and were subsequently averaged per kilogram of avoidable and possibly avoidable food waste, respectively. This was achieved by dividing the total nutrient values by the total amount of avoidable and possibly avoidable food waste, respectively. As an example of the nutrient losses from a single food item category, the nutrient content of the individual food item contributing the most to the preventable food waste fraction was calculated. Additionally, the nutrient contents were standardized to energy and expressed as nutrient density (per megajoule, MJ). To calculate this, the average nutrient values for the total waste of each fraction were divided by the corresponding total average energy contents of the waste: 1069.4 MJ, 133.0 MJ, and 86.3 MJ, respectively.

To perform the nutrition calculations for the avoidable and possibly avoidable food waste fractions, several assumptions were made. For categories containing various food items (e.g., citrus, leafy greens), average nutritional values were calculated based on common consumption options within each category. Additionally, assumptions were made regarding whether food items were in raw or cooked form (e.g., potato: 50% raw, 50% cooked). Lastly, due to the lack of data regarding the nutrient content of carrot and apple peels in fresh weight, the nutrient composition of carrot and apple with peel were used. A detailed breakdown of all categorized food groups and their assigned food items is provided in Tables S3 and S4 in the Supplementary material.

### Prevention potential scenarios

The potential impact of reducing food waste depends on the type of food wasted and the consequences of achieving such reductions. To explore this from different perspectives, two prevention scenarios were developed. In line with the SDG target 12.3, each scenario was based on a 50% reduction in food waste, assessed in terms of weight and the climate, economic, and nutritional impacts. Although the SDG target does not explicitly state whether it refers to total food waste or just the avoidable fraction, this study focused exclusively on the preventable fraction as this fraction represents the portion of food waste that can realistically be prevented.

The first scenario assumed that the household would consume more food to reduce food waste. In this case, 50% of the preventable fraction would be eaten by the household members in addition to their usual intake. This scenario involved the avoided impact of waste management (apart from the economic impact), while the impact of production and subsequent stages up to consumption remained. This scenario did not consider a potential rebound effect as the household would not save any money by consuming more food.

The second scenario also assumed that the household would consume the food instead of wasting it, however, this food would replace an equivalent amount of food that would have been otherwise purchased. This meant that 50% of the preventable fraction would not have been produced in the first place, thereby avoiding impacts from both production and waste management (still excluding potential cost savings in waste management). However, this scenario also considered the financial savings for households, as less food would need to be purchased. These savings could instead be spent on other goods or services, potentially introducing a so called rebound effect, i.e. the climate impact mitigation of waste reduction would be partially offset by increased consumption elsewhere^26^. Following Albizzati et al.^43^, an illustrative rebound effect of 38% was applied to this scenario. It should be noted that this estimate was derived using a slightly different approach than in the present study, for instance considering the whole economy. Consequently, the rebound effect should be interpreted as an approximate adjustment rather than a precise estimate.

## Results

Of the 919 kg of categorized food waste, 224 kg (24.4%) was categorized as avoidable, 67 kg (7.3%) as possibly avoidable, and 628 kg (68.3%) as unavoidable. This suggests a prevention potential of 31.7% amongst the 41 households across the 9843 quantification days. Based on the total recorded food waste from the participating households, the average food waste generated per person per day was 0.140 kg (min: 0.060, max: 0.290). This resulted in an average generation of 34 g of avoidable food waste per person per day, along with another 10 g of possibly avoidable food waste. In total, this meant a prevention potential of 44 g per person per day or 16 kg per year.

### Composition of waste fractions

The food category with the greatest contribution to the avoidable fraction in terms of weight was vegetables (33.9%), followed by grain-based foods (27.5%), and fruits (18.8%). Meanwhile, nuts and legumes as well as sweets contributed the least with 1.3% and 0.7% of the total weight of the avoidable fraction, respectively. The contribution to the avoidable fraction of each food category and their sub-categories is available in the Supplementary material (Table S5).

From the possibly avoidable fraction, more than half (53%) of the weight comprised of potato peels, which were also the largest contributor to the preventable fraction on an individual food item level. In the unavoidable fraction, 51% consisted of unavoidable parts of foods, while coffee and tea remains made up an additional 42%. The final 7% of the unavoidable fraction consisted of non-food items such as flowers, paper towels, and wrongly sorted items. The predominant contributing category to the unavoidable food waste was fruit. In total, half of the unavoidable parts of food consisted of banana peels, citrus peels, and apple cores. A summary of the composition of categories in terms of weight and their contribution to each food waste fraction is presented in Table 1.

**Table 1:** Distribution of recorded food waste by fraction and food waste category, showing the relative contribution of each category to both fraction and total food waste. The table presents the total weight per food waste category, along with its contribution to the corresponding fraction (%) and its contribution to the total amount of food waste (%). Within each fraction, categories are listed by recorded weight.

Category	Weight (kg)	Contribution to category (%)	Contribution to total food waste (%)
**Avoidable**	**224**	**100**	**24.4**
Vegetables	76.0	33.9	8.3
Grain-based food	61.5	27.4	6.7
Fruit	42.0	18.8	4.6
Composite dish	25.3	11.3	2.7
Animal-based food	11.4	5.1	1.2
Other food	3.3	1.5	0.4
Nuts & legumes	2.9	1.3	0.3
Sweets	1.5	0.7	0.2
**Possibly avoidable**	**67.4**	**100**	**7.3**
Potato peel	35.5	52.7	3.9
Peel and parts from other vegetables	27.8	41.2	3.0
Fruit peel (apples & pears)	4.1	6.1	0.4
**Unavoidable**	**628**	**100**	**68.3**
Unavoidable parts of food	320	51.0	34.8
Coffee & tea remains	264	42.0	28.7
Non-food items	41.0	6.5	4.5
Wrongly sorted items	3.2	0.5	0.3

### Food waste impact

The carbon footprint of the avoidable food waste amounted to an average 1.4 kg CO_2_e per kg of food waste. For the possibly avoidable fraction, the corresponding value was 0.28 kg CO_2_e per kg food waste. Based on a weighted average for both fractions (i.e. preventable food waste), the carbon footprint amounted to 1.2 kg CO_2_e per kg, meaning that, in total, the average daily carbon footprint per person was 0.052 kg CO_2_e, amounting to 19 kg CO_2_e annually.

The cost of the wasted avoidable food amounted to €4.4 per kg, equaling a cost of €0.15 per person per day. The corresponding cost for the possibly avoidable fraction was €2.6 per kg, or €0.03 per person per day. The weighted average cost for the two fractions combined was €4.0 per kg. Together, this resulted in an economic saving potential of about €0.18 per person per day, or approximately €66 per year for the preventable fraction.

On a food category level in the avoidable fraction, animal-based food waste had the highest relative contribution to the carbon footprint while being the fourth and fifth category contributing to cost and weight, respectively. Meanwhile, vegetables were the biggest contributor to both weight and cost but ranked fourth in terms of carbon footprint. In the possibly avoidable fraction, potato peel contributed the most to the overall weight but was second to peels and parts from other vegetables in terms of carbon footprint and cost. A summary of all categories’ contribution to carbon footprint and cost is presented in Table 2.

**Table 2:** Carbon footprint (CF) and economic impact of preventable food waste (FW) per food waste category, divided into avoidable and possibly avoidable fractions. For each food waste category, the table presents total weight (kg), total CF (kg CO₂e), and total cost (€), along with their relative contribution to the total CF and cost (in %), and the CF and cost per kilogram of food waste. Categories are listed in order or recorded weight within each fraction.

Category	Weight (kg)	CF (kg CO_2_e)	Contribution to fraction (CF-%)	CF per kg FW (kg CO_2_e)	Cost (€)	Contribution to fraction (cost-%)	Cost per kg FW (€)
**Avoidable**	**224**	**346**	**100**	**1.5**	**992**	**100**	**4.4**
Vegetables	76.0	59	17.0	0.8	367	37.0	4.8
Grain-based food	61.5	71	20.6	1.2	175	17.6	2.8
Fruit	42.0	33	9.6	0.8	152	15.3	3.6
Composite dish	25.3	61	17.6	2.4	121	12.2	4.8
Animal-based food	11.4	108	31.1	9.4	125	12.6	10.9
Other food	3.3	10	2.9	3.0	21	2.1	6.2
Nuts & legumes	2.9	1.8	0.5	0.6	20	2.0	6.7
Sweets	1.5	2.5	0.7	1.7	12	1.2	7.8
**Possibly avoidable**	**67.4**	**26**	**100**	**0.4**	**173**	**100**	**2.6**
Potato peel	35.5	10	40.2	0.3	65	37.4	1.8
Peel and parts from other vegetables	27.8	13	50.4	0.5	96	55.8	3.5
Fruit peel	4.1	2.4	9.4	0.6	12	6.8	2.9

The assessment of nutrient losses in avoidable and possibly avoidable waste revealed an energy content of 4.8 MJ and 2.0 MJ per kg of waste, respectively. The individual food item contributing the most to the preventable fraction in total, potato peels, had a corresponding energy content of 2.4 MJ per kg (Table 3). The protein, fat, and carbohydrate contents were 40 g, 28 g, and 168 g per kg of avoidable food waste, 20 g, 2 g, and 92 g per kg of possibly avoidable food waste, and for potato peel waste specifically, 26 g, 1 g, and 124 g per kg, respectively.

The analysis also indicated significant micronutrient losses, as all waste fractions contained high levels of various micronutrients. All waste fractions, avoidable, possibly avoidable, and potato peels (a sub-fraction within the possibly avoidable fraction), exceeded the recommended nutrient density for dietary planning^44^ for dietary fiber (3 g/MJ), folate (45 µg/MJ), and vitamin C (8 mg/MJ). The measured densities were 5.2, 12, and 10 g/MJ for dietary fiber; 60, 142, and 70 µg/MJ for folate; and 31, 98, and 47 mg/MJ for vitamin C, respectively (Table 3). In contrast, while the iron density of avoidable food waste (1.4 mg/MJ) was below the recommended 1.6 mg/MJ, the iron density of possibly avoidable food waste (9.8 mg/MJ), and specifically of potato peels (13 mg/MJ), exceeded this threshold. For further details on nutrient losses, see Table S6 in Supplementary materials.

**Table 3:** Energy, macro-, and micronutrient content of avoidable and possibly avoidable food waste fractions, including potato peels (a sub-fraction of possibly avoidable food waste). Values are presented as total nutrient content, per kilogram, and nutrient density per megajoule (MJ).

		Avoidable food waste			Possibly avoidable food waste			Potato peel		
Energy and macronutrients	Unit	Total	Per kg	Nutrient density per MJ	Total	Per kg	Nutrient density per MJ	Total	Per kg	Nutrient density per MJ
Energy	kcal	255,700	1140		31,750	471		20,590	580	
Energy	kJ	1,069,400	4780		133,000	1970		86,270	2430	
Protein	g	9050	40	9	1340	20	10	912	26	11
Fat	g	6280	28	6	104	2	0.8	36	1	0.4
Carbohydrate	g	37,540	168	35	6220	92	47	4400	120	51
**Micronutrients**										
Dietary fiber	g	5600	25	5	1598	24	12	888	25	10
Folate	µg	64,270	287	60	18,940	281	142	6040	170	70
Vitamin C	mg	32,850	147	31	13,020	193	98	4050	110	47
Iron	mg	1490	7	1	1301	19	10	1150	32	13

MJ = megajoules; kcal = kilocalories; kJ = kilojoules; g = grams; µg = micrograms; mg = milligrams.

### Effects of halving preventable food waste

The results of the categorization showed that the total amount of preventable food waste was 44 g per person per day (34 g avoidable and 10 g possibly avoidable), corresponding to an annual amount of 16 kg. Therefore, the scenarios involving a 50% reduction assumed that 22 g of food waste would be avoided per person per day (17 g avoidable and 5 g possibly avoidable food waste), or 8.0 kg per year.

#### Scenario 1: assuming consumption of food

If half of the preventable food waste was consumed (in addition to normal consumption) rather than wasted, 22 g less food would be wasted per person per day, resulting in a reduction of 8.0 kg per year. In terms of nutrients, this meant an addition to the daily food intake of 1.7% of the nutrients and energy per kilogram of avoidable food waste listed in Table 3, which is equivalent to approximately 19 kcal, 0.4 g of dietary fiber, and 5 µg of folate. Additionally, a further 0.5% of the values per kg from the possibly avoidable fraction listed in Table 3 would be consumed daily, providing an additional 2 kcal, 1.4 µg of folate, and 1 mg of vitamin C.

This scenario generated no cost savings for the household, however, an increase in carbon footprint was found. The average carbon footprint of preventable food waste was found to be 1.2 kg CO_2_e per kg of food waste, 0.10 kg CO_2_e of which was savings from anaerobic digestion. If half of the preventable waste was eaten these savings would only apply to the half still wasted, which resulted in a total carbon footprint of 0.054 kg CO_2_e per person per day, or approximately 20 kg CO_2_e per year. This corresponds to a total increase of 4% in carbon footprint for this scenario compared to current conditions.

#### Scenario 2: assuming avoided production

When the assumption was instead that the reduced food waste would not have been produced in the first place, impacts from both waste management and all supply chain stages before that were excluded. In terms of avoided nutrient losses, this meant no changes to the current situation except rather than the food and embedded nutrients not being wasted they were instead not produced. However, in terms of costs and carbon footprint, differences were found.

From a cost perspective, a generated saving of €0.09 per person per day was found, totaling to an annual saving of approximately €33 which equals half of the cost lost under current conditions. The savings in costs had a further implication for the carbon footprint. When accounting for a rebound effect of 38% for the avoided food waste, a reduction potential in terms of carbon footprint was reduced to 0.8 kg CO_2_e per kg food waste. The 50% of avoided food waste in this scenario was thereby found to generate a carbon footprint mitigation of 0.02 kg CO_2_e per person per day compared to current conditions. Combining the carbon footprint of the avoided half and the half still wasted, this scenario resulted in a net positive carbon footprint of 0.04 kg CO_2_e per person per day, totaling to approximately 13 kg CO_2_e per year. This equals a saving in carbon footprint of 6 kg CO_2_e per year (32%) compared to current conditions.

## Discussion

By drawing on long-term quantification data, this study assessed the composition of food waste from 41 Swedish households, determined its prevention potential, and analyzed the potential effects of achieving the target of halving food waste. Results showed that 31.7% of total food waste was classified as preventable, representing the proportion that could realistically be reduced. This preventable fraction corresponded to 16 kg of food waste per person annually, associated with a carbon footprint of 19 kg CO_2_e and an economic loss of €66. The nutrient density of the preventable food waste was high, exceeding the recommended levels for nutrients such as folate and dietary fiber, both of which are commonly deficient among the Swedish population^44^. A particularly striking finding was that the nutritional quality of the possibly avoidable fraction, notably potato peels, far exceeded recommendations for these nutrients and contained exceptionally high levels of vitamin C (47 mg/MJ) and iron (13 mg/MJ), indicating that nutritionally valuable food is routinely discarded.

### Contextualizing results

Food waste, particularly at the household level, is often portrayed as a critical issue within current food systems. Reducing food waste has therefore been recognized as a key strategy to make food systems more sustainable, with the SDGs proposing a 50% reduction at the consumer level. Since this target was introduced, governments and organizations worldwide have dedicated significant efforts to address this issue. However, the proposed target remains unclear on whether it refers to total food waste or only the fraction that can reasonably be avoided, as well as what the impact would be if a 50% reduction were achieved. According to the findings of this study, if halving food waste refers to total mass, eliminating preventable food waste alone would be insufficient and would require about one-third of the unavoidable fraction to also be eliminated. This does not present a realistic scenario as it would require the prevention of items such as banana peels and coffee grounds. If food system transformation is to be driven by consumer behavior change, expecting widespread consumption of inedible items such as banana peels is both impractical and inefficient compared to broader dietary shifts towards reduced animal-based and increased plant-based food consumption^2^.

A more feasible interpretation of the reduction target is therefore to focus on the preventable fraction. However, as the results show, the benefits of doing so may be limited, and depend on how food waste is reduced. Halving preventable food waste by consuming it in addition to normal consumption, as is the case in the first scenario, even increased climate impact and yielded no economic savings, suggesting that this approach is not viable. If food waste is instead halved by avoiding the food being produced in the first place, as suggested in the second scenario, an individual would save about 6 kg CO_2_e and €33 annually. In the Swedish context, where average consumption-based emissions are approximately 8 tons CO_2_e per year, of which 17% (1.4 tons CO_2_e) are food-related^45^, this would translate to a mere 0.5% reduction in food-related emissions. Thus, since the climate impact of the wasted food is modest to begin with, the potential reduction is correspondingly small. Similarly, the economic saving of €33 corresponds to about 1% of the annual per capita household food expenditure^46^, and less than 0.1% of the Swedish median income^47^. These comparisons suggest that both the climate and economic benefits of halving household food waste are modest. For most households, a financial saving of less than €3 per person per month, equivalent to the cost of a single ice cream, is likely too small to serve as a meaningful motivation for actively reducing food waste. Although economic incentives have been considered a leverage point for interventions^48,49^, the results of this study indicate that their potential may be overestimated. Moreover, when contrasted with the potential impact of dietary shifts^2,50^, the question arises about whether household food waste reduction should maintain its current level of attention or if there are other issues that should be prioritized.

However, it is important to consider that dietary change, particularly reduced meat consumption, is often challenging as food choices are deeply embedded in identity, culture, and social norms^51,52^. In contrast, food waste prevention typically triggers less resistance as it does not threaten core dietary identities and is often viewed as a practical issue^53,54^. This study demonstrated that food waste contained significant amounts of nutrients per kg, many of which are under-consumed among the Swedish population (e.g., folate, dietary fiber, and iron), suggesting a possible entry point for improving nutrient intake. However, to significantly contribute to nutrition, recovered foods would ideally replace other less nutrient-dense items in the diet rather than simply increase total intake as this may result in trade-offs related to carbon footprint and economic impact as shown in scenario 1. Moreover, reducing food waste through additional consumption could also pose a risk for metabolic food waste. Indeed, scenario 1 also showed a daily increase of 21 kcal per person which would translate to approximately 1 kg of weight gain per year, assuming that energy balance is not adjusted elsewhere^55,56^. This may appear minor, but if food waste prevention leads to additional consumption, as opposed to substitution, then even a seemingly small daily increase of 21 kcal per person is not nutritionally neutral especially if in a context of already rising overweight and obesity.

While avoidable food waste may often stem from external factors such as spoilage or lack of planning, the largest contributor to the preventable fraction on an individual food item level, potato peels, was likely a result of food preferences. As such, this may be easier to address through targeted interventions framed as food waste prevention rather than dietary change. Noteworthily, potato peels, which accounted for more than half of the possibly avoidable fraction, were especially rich in iron and vitamin C. Given the prevalence of iron deficiency among women of reproductive age^44^, and the role of vitamin C in enhancing non-heme iron absorption^57^, this nutritional combination could bear relevance for public health and warrants further attention.

It is important to acknowledge that this study reflects a Swedish context, where issues such as increased emissions from landfills and economic incentives to save food are smaller compared to other countries. In other settings with different food cultures, higher levels of food waste, and less optimized waste treatment, the problem may be more pronounced. For example, Liu et al.^58^ reported that Chinese households generated 0.25 kg (43%) of avoidable food waste per person per day, generating a carbon footprint of 1.17 kg CO_2_e. In the United Kingdom, Cooper et al.^59^ reported avoidable food waste to amount to 0.23 kg per person per day, contributing to 0.9 kg CO_2_e, while Antonelli et al.^60^ found that Italian households wasted 0.076 kg of avoidable food per person per day with a carbon footprint of 0.18 kg CO_2_e. These varying results underscore the need to contextualize assessments, as both the magnitude and impact of food waste are shaped by regional and systematic differences.

Within Sweden, the findings of this study align closely with national estimates. The national average of 56 kg of household food waste per person per year^24^ is comparable to the 51 kg found in this study which also accounted for zero-waste days. Likewise, the avoidable fraction at the national level (27%) matches the preventable fraction of 31.7% in this study, resulting in similar absolute amounts of about 15–16 kg per year. However, although the amounts of food waste are similar, the food waste composition differs between this study and what has been reported at the national level. For example, this study found only 5% of preventable food waste to be animal-based, whereas the report on the national level suggested considerably higher proportions with meat alone accounting for 12% of the avoidable food waste^61^. These differences partially explain why the carbon footprint reported at the national level was almost three times higher than that found in this study (55 kg CO_2_e per kg avoidable food waste compared to 19 kg CO_2_e), yet the reasons behind this remain unknown. Furthermore, national estimates of economic losses associated with food waste were also similar to those of this study, with this study finding an annual loss of €66 compared to €78 reported by the Swedish Food Agency^62^.

### Limitations

A notable limitation of the study is its potential to be generalized beyond the sample of participating households since they did not represent the general population. Despite this, the close alignment of the results with Swedish national estimates suggests that the findings may still offer relevant insights on a broader scale, even if they are limited to Sweden or countries with similar food cultures and waste management systems. Additionally, the results were presented as average values, implying an assumption that all households generated similar amounts and types of food waste. Given that overall food waste quantities varied substantially between households (averages between 0.060 and 0.290 kg per person per day), individual prevention potential likely differs which should be considered when designing reduction interventions.

Moreover, categorization of food waste in terms of what is considered avoidable remains a debated topic, lacking an established standard to guide decisions. In this study, decisions were made subjectively by the authors, which may have led to over- or underestimations of certain food waste fractions. To increase objectivity, a separate category for possibly avoidable food waste was included to account for items on the borderline between avoidable and unavoidable. It should also be noted that not all food waste generated in the households was categorized, therefore the categorized portion may not fully reflect overall food waste. However, as the categorized events were randomly selected and many households had been quantifying their food waste throughout the year, the results can be considered reasonably representative within the sample. Still, it should be borne in mind that the quantification system only captured waste disposed of in the organic waste bin, meaning that the quantified food waste possibly did not reflect overall food waste as some waste may have entered other waste streams. For instance, the low amount of animal-based food waste may be attributed to reasons such as these items not being separated from their packaging due to inconvenience, leaving them to be discarded in the residual waste bin.

Beyond the categorization, there are other methodological limitations that may have influenced the results. One limitation concerns the quantification technology used in the study, which is still under active development. Not all collected data could be used to assess food waste composition and its impacts, primarily due to poor image quality resulting from suboptimal installation conditions. Improving the system’s adaptability to different kitchen layouts would increase data coverage and reliability, which represents a key area for further technological development. Another methodological limitation is the calculation of the carbon footprint and the assumptions underlying it. For instance, both the carbon footprint values suggested in the SAFAD tool and the assumed rebound effect of 38% applied in the second scenario may have been either underestimated or overestimated. However, despite these uncertainties, the results provided an indication to the scale of impact associated with the food waste in the participating households. In the case of the rebound effect, even if excluding it entirely, the estimated annual carbon footprint reduction from halving the preventable fraction would increase from 6 to 9 kg CO_2_e, which is still a relatively minor saving.

### Moving forward

Although the findings of this study suggest that food waste in Swedish households may not pose a major barrier to sustainable food systems, substantial efforts and resources continue to be devoted to addressing the issue, both in Sweden and globally. However, given that environmental impacts, including carbon footprints, are likely greater outside of Sweden, especially in regions with less optimized waste management systems^3^, food waste should not be overlooked as global concern. Based on the study results, certain recommendations for future efforts can be proposed.

The results, which were based on the averages across the study participants, suggested that the overall impact of food waste was relatively limited. However, previous research has shown considerable variation in food waste generation both between and within households^32,63^. This highlights the need for more targeted analyses to identify household characteristics associated with higher prevention potential. While the sample size in this study did not allow for robust segmentation of households, future studies with larger datasets could apply such approaches to support the development of tailored interventions, rather than relying on generalized, one-size-fits-all approaches.

Additionally, international comparisons indicated that food waste quantities and associated carbon footprints are higher outside of Sweden. This emphasizes the importance of prioritizing food waste reduction in regions where the problem is more severe, and where a reduction could yield more significant benefits. In such cases, strategies aimed at mitigating the impact of food waste should also not solely focus on reducing food waste within households but should also strive to improve waste treatment infrastructure, such as by promoting anaerobic digestion or composting over landfilling.

Lastly, because the study findings suggest that the benefits of food waste reduction may be limited, at least within Sweden, questions arise about where effort and resources are best allocated to mitigate the impact of the food system. If food waste reduction alone may yield only minor benefits, this suggests that such initiatives should be integrated into broader strategies aimed at transforming the food system. This would involve moving beyond individual consumer behavior and instead adopting a broader system-level approach, which would necessitate engagement from policymakers and industry actors as they play a crucial role in shaping the conditions under which lasting behavioral change becomes feasible. Without structural support and engagement from all actors along the food supply chain, interventions that rely solely on consumers are unlikely to deliver meaningful results that can contribute to long-term changes^64^.

## Conclusions

The findings from this study indicated that 31.7% of total food waste amongst participating households was preventable, representing the fraction that can be realistically reduced. However, halving this fraction would yield only modest economic savings for the households, likely insufficient to serve as a strong behavioral motivator. It would also result in a relatively minor reduced carbon footprint. These results suggest that, in the Swedish context, prioritizing other strategies, such as promoting dietary shifts toward more sustainable food consumption patterns, may provide greater benefits. Nevertheless, the study also found that preventable food waste, particularly the possibly avoidable fraction, contained considerable nutritional values. This highlights that reducing household food waste could contribute to public health objectives by promoting the consumption of nutritionally valuable foods that are currently discarded. Overall, this study concludes that, although household food waste reduction can contribute to climate, economic, and nutrition benefits, its overall impact may be relatively limited within Sweden. Therefore, while efforts to reduce food waste should not be abandoned, greater emphasis should be placed on more impactful strategies, such as promoting sustainable dietary patterns.

## Supplementary Information

Below is the link to the electronic supplementary material.

Supplementary Material 1

## Data Availability

The data used and analyzed during this study are available from the corresponding author.
