# Supplementary material for: Quantifying the realistic reduction potential of food waste in Swedish households
Source: Sci Rep. 2026 Feb 2;16:4323. doi: 10.1038/s41598-026-37302-7 (PMC12864908; doi:10.1038/s41598-026-37302-7)
Supplement: Supplementary file 1 — Supplementary Material 1 [file 41598_2026_37302_MOESM1_ESM.docx]

Supplementary material

Table S1: Information about the households participating in the study, including household composition, age distribution, and data coverage.

| **Household** | **Members** | **Kids* (n)** | **Age group**** | **Days with quantification data (n)** | **Amount FW categorized (%)** |
| --- | --- | --- | --- | --- | --- |
| 1 | 4 | Yes (2) | 31-45 | 465 | 0.2 |
| 2 | 5 | Yes (3) | 46-60 | 325 | 25.3 |
| 3 | 2 | No | 18-30 | 115 | 40.9 |
| 4 | 3 | Yes (1) | 31-45 | 418 | 41.6 |
| 5 | 1 | No | 18-30 | 191 | 8.5 |
| 6 | 2 | No | 31-45 | 353 | 18.7 |
| 7 | 1 | No | 31-45 | 284 | 45.1 |
| 8 | 1 | No | 31-45 | 283 | 67.8 |
| 9 | 4 | Yes (2) | 46-60 | 351 | 30.6 |
| 10 | 4 | Yes (2) | 46-60 | 385 | 37.5 |
| 11 | 4 | Yes (2) | 31-45 | 251 | 4.1 |
| 12 | 2 | No | 31-45 | 222 | 26.6 |
| 13 | 2 | No | 31-45 | 145 | 33.0 |
| 14 | 4 | Yes (2) | 46-60 | 212 | 51.1 |
| 15 | 4 | Yes (2) | 46-60 | 305 | 1.5 |
| 16 | 2 | No | 60+ | 323 | 87.4 |
| 17 | 2 | No | 60+ | 134 | 2.0 |
| 18 | 2 | No | 31-45 | 158 | 30.7 |
| 19 | 3 | Yes (1) | 31-45 | 367 | 22.3 |
| 20 | 1 | No | 18-30 | 218 | 13.4 |
| 21 | 1 | No | 60+ | 169 | 22.2 |
| 22 | 1 | No | 31-45 | 72 | 67.4 |
| 23 | 2 | No | 31-45 | 244 | 44.1 |
| 24 | 2 | No | 31-45 | 247 | 52.7 |
| 25 | 3 | Yes (1) | 46-60 | 149 | 67.6 |
| 26 | 2 | No | 60+ | 94 | 23.3 |
| 27 | 2 | No | 60+ | 234 | 0.1 |
| 28 | 2 | No | 60+ | 172 | 12.3 |
| 29 | 2 | No | 46-60 | 422 | 9.4 |
| 30 | 1 | No | 18-30 | 61 | 61.8 |
| 31 | 4 | Yes (2) | 31-45 | 87 | 32.9 |
| 32 | 1 | No | 31-45 | 146 | 55.1 |
| 33 | 2 | No | 31-45 | 72 | 61.5 |
| 34 | 1 | No | 31-45 | 51 | 57.6 |
| 35 | 1 | No | 18-30 | 44 | 74.5 |
| 36 | 3 | Yes (1) | 31-45 | 36 | 46.8 |
| 37 | 2 | No | 18-30 | 323 | 6.6 |
| 38 | 2 | No | 46-60 | 423 | 10.5 |
| 39 | 3 | Yes (1) | 31-45 | 424 | 32.5 |
| 40 | 2 | No | 18-30 | 405 | 78.0 |
| 41 | 3 | No | 31-45 | 463 | 3.5 |
| *Between 1 and 18 years old  **Of adult household members | | | | | |

Table S2: Carbon footprint (kg Co_2_e per kg food in rounded figures) assigned each food category in the preventable food waste fraction, based on data from the SAFAD tool (Swedish University of Agricultural Sciences 2025).

| **Category** | **Foods included** | **kg CO_2_e per kg (SAFAD data)** |
| --- | --- | --- |
| Apple | Apple (Malus domesticus) | 0.59 |
| Avocado | Avocados (Persea americana) | 1.41 |
| Banana | Bananas (Musa × paradisica) | 0.71 |
| Beef | Roast beef | 40.9 |
|  | Minced meat | 36.7 |
| Bell pepper | Peppers (Capsicum annuum, var. grossum and var. longum) | 0.66 |
| Berries | Strawberries (Fragaria × ananassa) | 0.51 |
|  | Raspberries (Rubus idaeus) | 1.18 |
|  | Blueberries (Vaccinium corymbosum) | 1.28 |
| Bread (hard) | Crisp bread, rye wholemeal | 0.84 |
|  | Crisp bread, rye, light | 0.85 |
|  | Crisp bread, wheat, wholemeal | 2.39 |
| Bread (soft) | Wheat bread, white | 0.69 |
|  | Wheat bread, brown | 1.20 |
|  | Multigrain bread | 1.21 |
|  | Tortilla bread | 1.43 |
| Broccoli | Broccoli, cooked | 0.97 |
|  | Broccoli, raw | 0.79 |
| Cabbage | Head cabbage (red) | 0.61 |
|  | Head cabbage (white) | 0.46 |
| Carrot | Carrots (Daucus carota) | 0.23 |
| Cauliflower | Cauliflower (Brassica oleracea var. botrytis) | 0.79 |
| Cheese | Cheese, Brie | 6.47 |
|  | Cheese, Gruyere | 9.39 |
|  | Feta cheese | 5.69 |
| Chicken | Chicken meat (cooked) | 3.37 |
| Citrus | Oranges (Citrus sinensis) | 0.87 |
|  | Lemons (Citrus limon) | 0.97 |
| Composite dish | Herring | 4.30 |
|  | Beef stew | 11.6 |
|  | Potato gratin | 1.98 |
|  | Chicken stew | 2.35 |
|  | Curry stew | 1.61 |
|  | Lentil stew | 0.48 |
|  | Pork stew | 3.22 |
|  | Vegetable stew | 0.70 |
|  | Pizza | 3.97 |
|  | Bread (tortilla) | 1.43 |
|  | Hamburger | 22.6 |
|  | Bolognese sauce | 9.59 |
|  | Legumes | 0.52 |
|  | Minced meat | 30.9 |
|  | Rice porridge | 1.31 |
|  | Lasagne | 4.25 |
|  | Gratin with meat | 10.7 |
|  | Mashed potato | 0.71 |
|  | Rice | 1.34 |
|  | Legume patties | 0.83 |
|  | Bread (hamburger) | 1.19 |
|  | Wrap | 5.49 |
|  | Lentil lasagne | 2.23 |
|  | Pancake | 1.44 |
|  | Lentils | 0.38 |
|  | Vegetable gratin | 1.61 |
|  | Pasta | 0.63 |
|  | Yoghurt | 1.28 |
|  | Waffle | 2.24 |
|  | Pork pancake | 2.28 |
|  | Ham & Cheese sandwich | 4.81 |
|  | Pasta gratin | 1.71 |
|  | Potato | 0.89 |
|  | Porridge | 0.32 |
|  | Ajvar relish sauce | 0.83 |
|  | Leafy greens (spinach) | 0.72 |
|  | Fried potato and meat dish | 8.68 |
|  | Tomato | 1.17 |
|  | Lentil Bolognese | 0.89 |
|  | Hummus | 1.60 |
|  | Apple sauce | 0.78 |
|  | Potato patty | 0.72 |
|  | Apple pie | 1.29 |
|  | Oat rice | 0.40 |
|  | Spanish tortilla | 1.90 |
|  | Tomato sauce | 1.22 |
|  | Couscous salad | 0.80 |
|  | Pan fried fish | 5.81 |
|  | Taco remains | 3.91 |
|  | Carrot | 0.23 |
|  | Onion | 0.33 |
|  | Other carbs | 0.81 |
|  | Corn | 1.04 |
|  | Crème fraiche | 3.76 |
|  | Falafel | 0.56 |
|  | Vegetable sauce | 0.70 |
|  | Lentil/dairy sauce | 0.70 |
|  | Salad | 0.92 |
|  | Dairy/vegetable sauce | 0.89 |
|  | Potato & ham gratin | 2.70 |
|  | Sausage stroganoff | 5.43 |
|  | Chickpeas | 0.22 |
|  | Bell pepper | 0.66 |
|  | Tuna fish salad | 1.90 |
|  | Peas | 0.25 |
|  | Green beans | 0.94 |
|  | Pesto | 3.09 |
|  | Feta cheese | 5.69 |
|  | Shrimp sauce | 8.60 |
| Cucumber | Cucumbers (Cucumis sativus) | 1.03 |
| Egg | Whole egg, chicken | 1.37 |
| Fish | Salmon (Cooked) | 8.26 |
| Leafy greens | Spinach (fresh) (Spinacia oleracea) | 0.72 |
|  | Iceberg-type lettuce | 0.54 |
|  | Kale (Brassica oleracea convar. Acephalea) | 0.46 |
| Legumes | Beans (dry), soaked/boiled/canned | 0.48 |
|  | Peas (dry), soaked/boiled/canned | 0.25 |
|  | Chick pea (Cicer arietinum) | 0.22 |
| Melon | Melons (Cucumis melo) | 0.71 |
| Mixed vegetables | Average of vegetables | 0.76 |
| Mixed fruit | Average of fruits | 0.67 |
| Nuts | Nuts, mixed | 2.13 |
| Onion | Onions, bulb (Allium cepa) | 0.33 |
|  | Leek (Allium porrum) | 0.48 |
| Other dairy | Crème fraîche | 3.76 |
|  | Yoghurt, cow milk, 1 - 3% fat | 1.28 |
|  | Cream 40 % fat | 3.76 |
| Other fruit | Pear (Pyrus communis) | 0.63 |
|  | Mangoes (Mangifera indica) | 0.85 |
|  | Table grapes (Vitis euvitis) | 1.21 |
|  | Pineapples (Ananas comosus) | 0.87 |
| Other vegetables | Sweet corn (Zea mays var. saccharata) | 1.04 |
|  | Table olives (Olea europaea) | 1.53 |
|  | Cultivated mushroom (syn. Button mushroom) | 3.86 |
|  | Aubergines (egg plants) (Solanum melongena) | 0.51 |
| Other/unknown carbs | Popcorn | 1.41 |
|  | Oatmeal, whole grain | 0.32 |
|  | Quinoa grain | 3.10 |
|  | Couscous | 0.54 |
| Other/unknown food | Average of all foods | 2.93 |
| Other/unknown meat | Falukorv (common Swedish sausage) | 7.48 |
|  | Mixed beef and pork mince | 30.9 |
|  | Meatballs | 15.7 |
| Pasta | Plain pasta, cooked | 0.63 |
| Pork | Ham, pork | 7.29 |
|  | Pork / piglet meat (Sus scrofa) | 7.51 |
|  | Bacon | 7.40 |
| Potato | Potato boiled | 0.89 |
|  | Potato fried | 0.65 |
|  | Potato baked | 0.51 |
|  | Mashed potatoes (fresh) | 0.71 |
| Pumpkin | Pumpkins (Cucurbita maxima) | 0.92 |
| Rice | Rice, cooked, polished | 1.34 |
| Root vegetables | Beetroot (Beta vulgaris subsp. vulgaris) | 0.23 |
|  | Parsnips (Pastinaca sativa) | 0.23 |
| Semi-fluids | Guacamole | 1.35 |
|  | Crème fraiche | 3.76 |
|  | Brown sauce | 1.46 |
|  | Jam | 1.20 |
|  | Ketchup | 2.61 |
|  | Hummus | 1.60 |
|  | Tomato sauce (pasta sauce) | 3.54 |
|  | Yoghurt | 1.28 |
|  | Sweet chili sauce | 0.62 |
|  | Pesto | 3.09 |
|  | Sun-dries tomatoes | 16.5 |
|  | Salad with dairy sauce | 2.93 |
| Sweets | Sweet bread, cinnamon bun, homemade | 1.65 |
|  | Candy | 1.32 |
|  | Sponge cake | 1.71 |
| Tomato | Tomato | 1.17 |
| Uncertain fruits | Average of all fruits | 0.80 |
| Uncertain vegetables | Average of all vegetables | 0.80 |
| Zucchini | Zucchini | 0.58 |

Table S3: Quantities of each food waste category and their subcategories, along with the assumed corresponding item(s) used to calculate the nutrient composition of the avoidable food waste fraction.

| **Category** | **Sub-category** | **Weight (kg)** | **Weight (g)** | **Comments** |
| --- | --- | --- | --- | --- |
| Beef | Beef | 0.14 | 137 | cooked |
| Bread (hard) | Bread (hard) | 3.83 | 3827 | husman |
| Bread (soft) | Bread (soft) | 19.99 | 19986 | 50% white; 50% whole grain |
| Cheese | Cheese | 4.90 | 4900 | 28% fat |
| Chicken | Chicken | 0.31 | 312 | cooked |
| Composite dish | Composite dish | 25.28 | 27453 | 10%: yoghurt with muesli; pyttipanna; lasagna;  stew; with lentils; pasta Bolognese; vegetarian lasagna; pizza; oatmeal; rice and chicken; potato gratin |
| Egg | Egg | 0.64 | 645 | cooked |
| Fish | Fish | 0.47 | 470 | salmon |
| Fruit | Apple | 15.16 | 15162 |  |
| Fruit | Banana | 3.76 | 3758 |  |
| Fruit | Berries | 3.20 | 3205 | 50% blueberries; 50% strawberries |
| Fruit | Citrus | 11.6 | 11549 | 33% orange; 33% mandarin; 33% lemon |
| Fruit | Melon | 3.10 | 3103 | 50% watermelon; 50% honey melon |
| Fruit | Other fruit | 4.25 | 4245 | 25% kiwi; 25% pear; 25% plum; 25% grapes |
| Fruit /veg | Mixed | 8.26 | 8261 | 50% mixed veg; 50% mixed fruit |
| Legumes | Legumes | 2.63 | 2626 | 50% white beans; 50% soy beans |
| Nuts | Nuts | 0.30 | 302 | 50% peanuts; 50% cashew |
| Other dairy | Other dairy | 2.38 | 2381 | 50% yoghurt; 50% sour cream |
| Other/unknown carbs | Other/unknown carbs | 10.5 | 10520 | 25% oatmeal; 25% couscous; 25% popcorn; 25% quin |
| Other/unknown food | Other/unknown food | 2.17 | 0 | calculated together with composite dish |
| Other/unknown meat | Other/unknown meat | 1.55 | 1554 | 33% sausage; 33% meatballs; 33% minced meat (cooked) |
| Pasta | Pasta | 15.8 | 15814 | cooked |
| Pork | Pork | 0.98 | 975 | cooked |
| Potato | Potato | 14.9 | 14894 | 50% raw; 50% cooked |
| Rice | Rice | 11.4 | 11362 | cooked |
| Semi-fluids | Semi-fluids | 1.17 | 1174 | 33% tomato sauce; 33% yoghurt; 33% brown sauce |
| Sweets | Sweets | 1.49 | 1495 | cinnamon buns |
| Veggies | Avocado | 1.82 | 1818 |  |
| Veggies | Bell pepper | 1.41 | 1413 |  |
| Veggies | Broccoli | 1.00 | 996 |  |
| Veggies | Cabbage | 12.7 | 12710 |  |
| Veggies | Carrot | 3.46 | 3457 |  |
| Veggies | Cauliflower | 1.20 | 1204 |  |
| Veggies | Cucumber | 4.72 | 4716 |  |
| Veggies | Leafy greens | 11.5 | 11532 | 33% iceberg salad; 33% spinach; 33% arugula |
| Veggies | Onion | 5.94 | 5936 | yellow onion |
| Veggies | Other vegetables | 3.39 | 3389 | 25% mushroom; 25% green peas; 25% leak; 25% lentils |
| Veggies | Pumpkin | 0.79 | 786 |  |
| Veggies | Root vegetables | 0.82 | 823 | 50% beetroot; 50% parsnip |
| Veggies | Tomato | 4.08 | 4084 |  |
| Veggies | Zucchini | 0.93 | 927 |  |
| **Total** |  | **224** | **223897** |  |

Table S4: Quantities of each food waste category and their subcategories, along with the assumed corresponding item(s) used to calculate the nutrient composition of the possibly avoidable food waste fraction.

| **Category** | **Sub-category** | **State** | **Weight (kg)** | **Weight (g)** | **Comments** |
| --- | --- | --- | --- | --- | --- |
| Potato | Potato | Peel | 35.424 | 35424 | nutrients retrieved from usda.com |
| Potato | Potato | Borderline items | 0.077 | 77 | nutrients retrieved from usda.com |
| veggies | broccoli | Borderline items | 2.622 | 2622 | broccoli |
| Veggies | Cabbage | Borderline items | 3.210 | 3210 | cabbage |
| Veggies | Carrot | Peel | 10.035 | 10035 | carrot |
| Veggies | Carrot | Borderline items | 0.017 | 17 | carrot |
| Veggies | Cauliflower | Borderline items | 1.513 | 1513 | cauliflower |
| Veggies | Cucumber | Peel | 0.553 | 553 | cucumber |
| Veggies | Cucumber | Borderline items | 0.148 | 148 | cucumber |
| Veggies | Leafy greens | Borderline items | 2.419 | 2419 | 50% spinach, 50% arugula |
| Veggies | Mixed | Borderline items | 1.022 | 1022 | 20% potato; 20% carrot; 20% tomato; 20% bell pepper; 20% broccoli |
| Veggies | Mixed | Peel | 1.442 | 1442 | 20% potato; 20% carrot; 20% tomato; 20% bell pepper; 20% broccoli |
| Veggies | Other vegetables | Borderline items | 0.433 | 433 | 20% potato; 20% carrot; 20% tomato; 20% bell pepper; 20% broccoli |
| Veggies | Other vegetables | Peel | 0.544 | 544 | 20% potato; 20% carrot; 20% tomato; 20% bell pepper; 20% broccoli |
| Veggies | Pumpkin | Borderline items | 2.649 | 2649 | pumpkin |
| Veggies | Root vegetables | Peel | 1.028 | 1028 | 20% potato; 20% carrot; 20% tomato; 20% bell pepper; 20% broccoli |
| Veggies | Tomato | Borderline items | 0.024 | 24 | 20% potato; 20% carrot; 20% tomato; 20% bell pepper; 20% broccoli |
| Veggies | Uncertain | Peel | 0.018 | 18 | 20% potato; 20% carrot; 20% tomato; 20% bell pepper; 20% broccoli |
| Veggies | Veggies | Borderline items | 0.125 | 125 | 20% potato; 20% carrot; 20% tomato; 20% bell pepper; 20% broccoli |
| Fruit | Apple | Peel | 1.549 | 1549 | Apple with skin |
| Fruit | Apple | Borderline items | 2.426 | 2426 | Apple with skin |
| Fruit | Other fruit | Peel | 0.072 | 72 | Apple with skin |
| Fruit | Other fruit | Borderline items | 0.053 | 53 | Apple with skin |
| Other/unknown food | Other/unknown food | Borderline items | 0.005 | 5 | Apple with skin |
| Other/unknown food | Other/unknown food | Peel | 0.009 | 9 | Apple with skin |
| **Total** |  |  | **67.4** | **67414** |  |

Table S5: Number of recorded events (n) and the weight (kg) of each food waste category and their sub-categories in the avoidable food waste fraction.

| **Main category** | **Sub-categories** | **Recorded events (n)** | **Recorded weight (kg)** |
| --- | --- | --- | --- |
| **Animal-based food** | Cheese | 137 | 4.98 |
|  | Other dairy | 40 | 2.38 |
|  | Other /unknown meat | 25 | 1.55 |
|  | Pork | 22 | 0.98 |
|  | Egg | 13 | 0.65 |
|  | Fish and shellfish | 5 | 0.47 |
|  | Chicken | 7 | 0.31 |
|  | Beef | 7 | 0.14 |
| **Composite dish** | Composite dishes (full list available in Table S1) | 329 | 25.28 |
| **Fruit** | Apple | 131 | 15.16 |
|  | Citrus | 126 | 11.55 |
|  | Other fruits | 62 | 4.30 |
|  | Banana | 35 | 3.76 |
|  | Berries | 56 | 3.21 |
|  | Melon | 20 | 3.10 |
|  | Mixed | 6 | 0.95 |
| **Grain-based food** | Bread (soft) | 438 | 19.99 |
|  | Pasta | 229 | 15.81 |
|  | Rice | 163 | 11.36 |
|  | Other/unknown carbs | 190 | 10.52 |
|  | Bread (hard) | 55 | 3.83 |
| **Nuts and legumes** | Legumes | 54 | 2.63 |
|  | Nuts | 11 | 0.30 |
| **Other food** | Other/unknown food | 37 | 2.17 |
|  | Semi-fluids | 21 | 1.17 |
| **Sweets** | Sweets (such as sweet bread and candy) | 34 | 1.50 |
| **Vegetables** | Potato | 160 | 14.89 |
|  | Cabbage | 104 | 12.71 |
|  | Leafy greens | 235 | 11.53 |
|  | Mixed | 100 | 7.24 |
|  | Onion | 127 | 5.94 |
|  | Cucumber | 95 | 4.72 |
|  | Tomato | 110 | 4.08 |
|  | Other vegetables | 68 | 3.48 |
|  | Carrot | 40 | 3.46 |
|  | Avocado | 26 | 1.82 |
|  | Bell pepper | 33 | 1.41 |
|  | Cauliflower | 9 | 1.20 |
|  | Broccoli | 8 | 1.00 |
|  | Zucchini | 7 | 0.93 |
|  | Other root vegetables | 7 | 0.82 |
|  | Pumpkin | 5 | 0.79 |
| **Total** |  | **3387** | **224** |

Table S6: The energy, macronutrient and micronutrient contents of the avoidable and possibly avoidable fractions, as well of potato peels (a sub-fraction of possibly avoidable food waste) which on an individual food item level contributed the most to the preventable food waste fraction. Values are presented as total nutrient content, per kilogram, and nutrient density per megajoule (MJ).

| **Energy and macro nutrients** | **Unit** | **Avoidable food waste** | | | **Possibly avoidable food waste** | | | **Potato peel** | | |
| --- | --- | --- | --- | --- | --- | --- | --- | --- | --- | --- |
|  |  | **Total** | **Per kg** | **Nutrient density per MJ** | **Total** | **Per kg** | **Nutrient density per MJ** | **Total** | **Per kg** | **Nutrient density per MJ** |
| Energy | kcal | 255701 | 1142 |  | 31749 | 471.0 |  | 20590 | 580 |  |
| Energy | kJ | 1069406 | 4776 |  | 133026 | 1973 |  | 86265 | 2430 |  |
| Protein | g | 9053 | 40.4 | 8.5 | 1344 | 19.9 | 10.1 | 912 | 25.7 | 10.6 |
| Fat | g | 6275 | 28.0 | 5.9 | 104 | 1.5 | 0.8 | 36 | 1 | 0.4 |
| Carbohydrate | g | 37536 | 167.6 | 35.1 | 6223 | 92.3 | 46.8 | 4402 | 124 | 51.0 |
| **Micronutrients** |  |  |  |  |  |  |  |  |  |  |
| Dietary fiber | g | 5602 | 25.0 | 5.2 | 1598 | 23.7 | 12.0 | 888 | 25 | 10.3 |
| Saturated fats | g | 2261 | 10.1 | 2.1 | 29.9 | 0.4 | 0.2 | 11 | 0.3 | 0.1 |
| MUFA | g | 2197 | 9.8 | 2.1 | 4.3 | 0.1 | 0.0 | 1 | 0.02 | 0.0 |
| PUFA | g | 989 | 4.4 | 0.9 | 46.3 | 0.7 | 0.3 | 14 | 0.4 | 0.2 |
| Vitamin A | RE | 91941 | 411 | 86 | 105215 | 1561 | 791 | 0 | 0 | 0.0 |
| Vitamin D | μg | 236 | 1.1 | 0.2 | 0.0 | 0.0 | 0.0 | 0 | 0 | 0.0 |
| Vitamin E | mg | 1372 | 6.1 | 1.3 | 224.5 | 3.3 | 1.7 |  |  |  |
| Vitamin K | μg | 35230 | 157.3 | 32.9 | 12258 | 181.8 | 92.1 |  |  |  |
| Vitamin B1 | mg | 205 | 0.9 | 0.2 | 25.9 | 0.4 | 0.2 | 7 | 0.2 | 0.1 |
| Vitamin B2 | mg | 158 | 0.7 | 0.1 | 27.7 | 0.4 | 0.2 | 14 | 0.4 | 0.2 |
| Niacin | NE | 3991 | 17.8 | 3.7 | 630.6 | 9.4 | 4.7 | 366 | 10.3 | 4.2 |
| Vitamin B6 | mg | 280 | 1.2 | 0.3 | 125.5 | 1.9 | 0.9 | 85 | 2.4 | 1.0 |
| Folate | μg | 64271 | 287 | 60 | 18943 | 281 | 142 | 6035 | 170 | 70 |
| Vitamin B12 | μg | 235 | 1.1 | 0.2 | 0.0 | 0.0 | 0.0 | 0 | 0 | 0.0 |
| Vitamin C | mg | 32849 | 147 | 31 | 13023 | 193 | 98 | 4047 | 114 | 47 |
| Calcium | mg | 119042 | 532 | 111 | 21551 | 320 | 162 | 10650 | 300 | 124 |
| Phosphor | mg | 182412 | 815 | 171 | 23765 | 353 | 179 | 13490 | 380 | 156 |
| Iron | mg | 1490 | 6.7 | 1.4 | 1301 | 19.3 | 9.8 | 1150 | 32.4 | 13.3 |
| Zinc | mg | 1415 | 6.3 | 1.3 | 201.7 | 3.0 | 1.5 | 124 | 3.5 | 1.4 |
| Selenium | μg | 3806 | 17.0 | 3.6 | 296.7 | 4.4 | 2.2 | 107 | 3 | 1.2 |
| **Abbreviations:** kcal = kilocalories; kJ = kilojoules; MJ = megajoules; g = grams; RE = retinol equivalents; μg = micrograms; mg = milligrams; NE = niacin equivalents. | | | | | | | | | | |

# References

Swedish University of Agricultural Sciences (2025). *SAFAD - Sustainability Assessment of Food and Diets* (1.262). https://safad.se/
